# Ribosome dynamics during decoding

**DOI:** 10.1098/rstb.2016.0182

**Published:** 2017-03-19

**Authors:** Marina V. Rodnina, Niels Fischer, Cristina Maracci, Holger Stark

**Affiliations:** 1 Department of Physical Biochemistry, Max Planck Institute for Biophysical Chemistry, Am Fassberg 11, Göttingen 37077, Germany; 2 Department of Structural Dynamics, Max Planck Institute for Biophysical Chemistry, Am Fassberg 11, Göttingen 37077, Germany

**Keywords:** ribosome, tRNA, translation, decoding, recoding

## Abstract

Elongation factors Tu (EF-Tu) and SelB are translational GTPases that deliver aminoacyl-tRNAs (aa-tRNAs) to the ribosome. In each canonical round of translation elongation, aa-tRNAs, assisted by EF-Tu, decode mRNA codons and insert the respective amino acid into the growing peptide chain. Stop codons usually lead to translation termination; however, in special cases UGA codons are recoded to selenocysteine (Sec) with the help of SelB. Recruitment of EF-Tu and SelB together with their respective aa-tRNAs to the ribosome is a multistep process. In this review, we summarize recent progress in understanding the role of ribosome dynamics in aa-tRNA selection. We describe the path to correct codon recognition by canonical elongator aa-tRNA and Sec-tRNA^Sec^ and discuss the local and global rearrangements of the ribosome in response to correct and incorrect aa-tRNAs. We present the mechanisms of GTPase activation and GTP hydrolysis of EF-Tu and SelB and summarize what is known about the accommodation of aa-tRNA on the ribosome after its release from the elongation factor. We show how ribosome dynamics ensures high selectivity for the cognate aa-tRNA and suggest that conformational fluctuations, induced fit and kinetic discrimination play major roles in maintaining the speed and fidelity of translation.

This article is part of the themed issue ‘Perspectives on the ribosome’.

## 1. Decoding and recoding

Translation of the genetic information is one of the fundamental processes in living cells. In each round of translation elongation, mRNA triplets are decoded by aminoacyl-tRNAs (aa-tRNAs) which are delivered to the A site of the ribosome in the complex with elongation factor Tu (EF-Tu; in bacteria, or eEF1A in eukaryotes) and GTP. EF-Tu and eEF1A bring all canonical elongator aa-tRNA to the A site where they decode sense codons of the mRNA. However, one uncommon amino acid, selenocysteine (Sec), which is carried by tRNA^Sec^, requires a specialized translation factor, SelB in bacteria or its homologue eEFSec in eukaryotes [1,2]. Sec-tRNA^Sec^ recodes a stop codon UGA in a programmed fashion: in bacterial mRNAs, the stop codon reassigned for Sec is followed by a hairpin structure known as selenocysteine insertion sequence (SECIS) [1]. Recoding of UGA by Sec, as well as of another stop codon UAG by pyrrolysine are *bona fide* recoding events that occur in the A site of the ribosome. By contrast, other types of ‘recoding’, such as frameshifting and bypassing, appear to be more related to tRNA–mRNA translocation from the A to P and P to E sites or the sliding of the ribosome along the mRNA [3–6]. These phenomena are considered ‘recoding’ in the sense that the information coded by the mRNA is read in an alternative way, but do not involve unconventional codon reading in the A site. In this review, we focus on the EF-Tu- and SelB-mediated decoding/recoding at the A site.

EF-Tu and SelB are translational GTPases that share homologous domains 1–3; SelB has an additional domain 4 that binds to the SECIS [7]. Despite the similar function, the nucleotide-binding properties of EF-Tu and SelB are quite different. EF-Tu binds GDP tighter than GTP and requires a nucleotide exchange factor, EF-Ts, to regenerate the active form of the GTPase [8], whereas SelB has an intrinsically high affinity for GTP and does not need a nucleotide exchange factor [9,10]. Both factors in their GTP form interact with their respective aa-tRNAs. Because SelB has evolved to bind a single aa-tRNA, it can recognize Sec and tRNA^Sec^ by a network of precisely tuned interactions [11–13], resulting in a very high binding affinity, 0.2 pM [9]. By contrast, EF-Tu, which has to bind different aa-tRNAs, employs an affinity compensation mechanism that relies on the relative contributions to binding of the amino acid and tRNA and leads to a uniform overall affinity of about 10 nM regardless of aa-tRNA [14,15].

The main challenge in understanding the decoding process is to uncover the mechanisms that ensure the high fidelity of decoding. Ultimately, the information on which aa-tRNA is cognate at a given cycle of translation elongation is defined by the complementarity between the mRNA codon and the anticodon of the aa-tRNA. However, it has long been recognized that the difference in the stability of correct and incorrect base pairs is insufficient to ensure the observed high fidelity of decoding and, furthermore, even the existing modest discrimination potential cannot be used in full due to the high speed of translation [16–18]. Thus, the complementarity of the codon–anticodon interaction must be sensed by the ribosome in some additional ways. We have shown that the ribosome employs the kinetic discrimination mechanism to select correct aa-tRNA from the bulk of cognate, near-cognate and non-cognate substrates and that the rates of EF-Tu GTPase activation and aa-tRNA accommodation in the A site are greatly accelerated when the anticodon of aa-tRNA matches the codon in the A site [17,19]. However, the structural basis of aa-tRNA selection remained poorly understood and in particular the mechanism of codon-dependent GTPase activation has been unclear for a long time. Pioneering work by Ramakrishnan and co-workers [20,21] has demonstrated how the ribosome recognizes correct codon–anticodon complexes. They showed that cognate codon–anticodon base pairing induces a local conformational change in the decoding centre of the small ribosomal subunit (SSU), where two universally conserved adenines, A1492 and A1493, change their position from the ‘flipped-in’ arrangement, pointing away from the mRNA codon, towards ‘flipped-out’ oriented towards the codon–anticodon complex. The two key adenines—with the help of several other residues at the decoding site—recognize the Watson–Crick geometry of the correct base pairs. The local rearrangement at the decoding centre leads to the global closure of the SSU head and body domains. Conformational dynamics of the SSU is crucial for the GTPase activation of EF-Tu and the subsequent aa-tRNA accommodation in the A site on the large ribosomal subunit (LSU) [22,23], suggesting an important role of ribosome dynamics and induced fit in tRNA selection. Our recent reconstruction of several intermediates on the route to UGA decoding by SelB–Sec-tRNA^Sec^ finally provides insights into the mechanism of how correct codon–anticodon recognition facilitates GTPase activation [13]. These concepts provide explanations for numerous existing observations and will stimulate new experiments and development of novel approaches to study decoding. In the following, we will summarize the current views on how the ribosome selects cognate aa-tRNA for translation.

## 2. The path to correct codon recognition

EF-Tu-mediated aa-tRNA delivery to the ribosome proceeds through a number of steps. The initial identification of several discrete states on the EF-Tu pathway was carried out by biochemical methods and ensemble kinetics [19]. Single molecule techniques suggested the existence of additional transient steps and underlined the importance of dynamic fluctuations [24]. The structures of several late intermediates have been solved by X-ray crystallography or cryo-electron microscopy (EM) [25–27]. Molecular dynamics simulations have been successful in modelling the detailed trajectories of motions along the tRNA accommodation pathway [28]. Finally, genetic analysis provided powerful tools to probe the interactions at each state [29–32], leading to a rather consistent picture of what happens during decoding.

**Figure 1.**
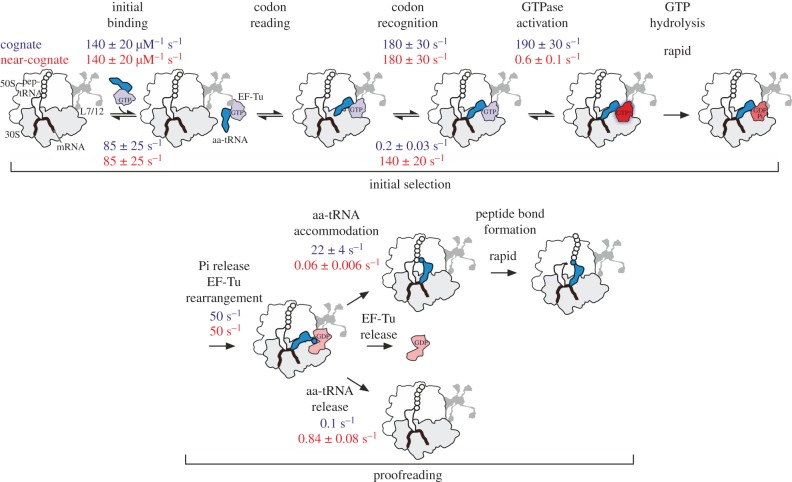
Schematic of the EF-Tu-dependent aa-tRNA delivery to the A site. The decoding pathway comprises two subsequent selection steps, initial selection and proofreading. The sequence of steps is based on ensemble kinetics and single molecule FRET experiments. Rate constants of kinetically important steps are shown for cognate (blue) and near-cognate (red) aa-tRNAs at 20°C [17,33–35]. The rate constants of the two irreversible steps, GTP hydrolysis and peptide bond formation, are limited by the respective preceding steps.

The sequence of major steps in aa-tRNA delivery to the ribosome (figure 1) starts with the initial binding of EF-Tu–GTP–aa-tRNA [19,30,36], followed by attempts of the tRNA to read the codon [37,38]. After codon recognition, the GTPase of EF-Tu is activated leading to GTP hydrolysis [33,34,39–41]. GTP hydrolysis in EF-Tu, like in all other translational GTPases, requires docking of the GTP-binding domain onto the sarcin–ricin loop (SRL) of the LSU. The subsequent Pi release [42] and the rearrangement of EF-Tu into the GDP-bound conformation releases aa-tRNA from the factor. The tRNA moves to accommodate itself into the A site of the peptidyl transferase centre, whereas EF-Tu–GDP dissociates from the ribosome [40,43]. Each of these major steps most probably includes additional rearrangements and movements [43,44] which are often too rapid to be resolved. The sequence of events for the eukaryotic analogue of EF-Tu, eEF1A, is probably similar [45,46].

**Figure 2.**
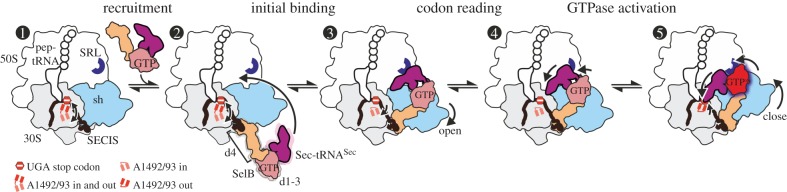
Mechanism of UGA recoding to Sec by SelB–Sec-tRNA^Sec^ as delineated by cryo-EM. Labels: pep-tRNA, peptidyl-tRNA; d, domains of SelB; dark red shading, GTPase-activated state of SelB; arrows ‘open’ and ‘close’, domain opening and closure of the 30S shoulder (sh). States 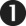 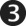 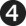 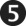 correspond to structural intermediates resolved by cryo-EM; state 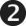 was modelled based on the structural data [13]. 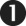 The initial complex contains an mRNA with a UGA stop codon in the A site and a SECIS element exposed for SelB recruitment. The ribosome is in the classical state with a peptidyl-tRNA in the P site and a vacant A site, while the universally conserved bases A1492 and A1493 of 16S rRNA fluctuate between the flipped-in and flipped-out conformations. 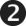 The recruitment state is formed upon binding of SelB–GTP–Sec-tRNA^Sec^ to the SECIS. The contact between SelB domain 4 and the SECIS is maintained in all subsequent steps. Sec-tRNA^Sec^ can spontaneously sample a broad range of different conformations. 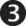 In the transient initial binding state, Sec-tRNA^Sec^ binds to the SRL and SelB to the shoulder domain of the 30S subunit. The shoulder domain moves apart stabilizing A1492 and A1493 in a flipped-in conformation. 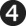 The distance to the UGA codon decreases as tRNA^Sec^ attempts to read the codon in the transient codon reading state. 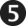 GTPase-activated pre-hydrolysis state. Codon recognition by tRNA^Sec^ induces a local closure of the decoding centre with A1492 and A1493 flipping out. The resulting global closure of the shoulder domain leads to repositioning of the tRNA and docking of SelB on the SRL. The docking leads to a codon-dependent GTPase activation of SelB (dark red shading) and GTP hydrolysis. Notably, SelB, which initially interacts with the 30S shoulder only, does not interact with the SRL until the final GTPase-activated state is reached.

The pathway for the specialized Sec delivery complex, SelB–GTP–Sec-tRNA^Sec^, starts with its recruitment to the SECIS. Because SelB–GTP–Sec-tRNA^Sec^ binds to the isolated SECIS very rapidly and tightly [10], the ternary complex binds to the SECIS even before the translating ribosome has arrived at the SECIS (figure 2). When the ribosome approaches the UGA codon during translation, SECIS-tethered SelB occupies a strategic position across the intersubunit space, blocking the approach to the A site of the release factor 2 (RF2) which normally reads the UGA codon, providing an explanation of why stop codon recognition by SelB does not compete with termination [47]. In the next steps, SelB has to dock onto the SRL of the LSU and hydrolyse GTP. Similarly to EF-Tu, GTP hydrolysis by SelB is necessary to release the tRNA from the factor prior to accommodation and peptide bond formation [9,48]. At all steps on the way to decoding, the ribosome, the elongation factor and aa-tRNA change their positions and conformations, and in the following we will discuss the dynamics of these changes from state to state.

## 3. Initial binding and codon sampling

**Figure 3.**
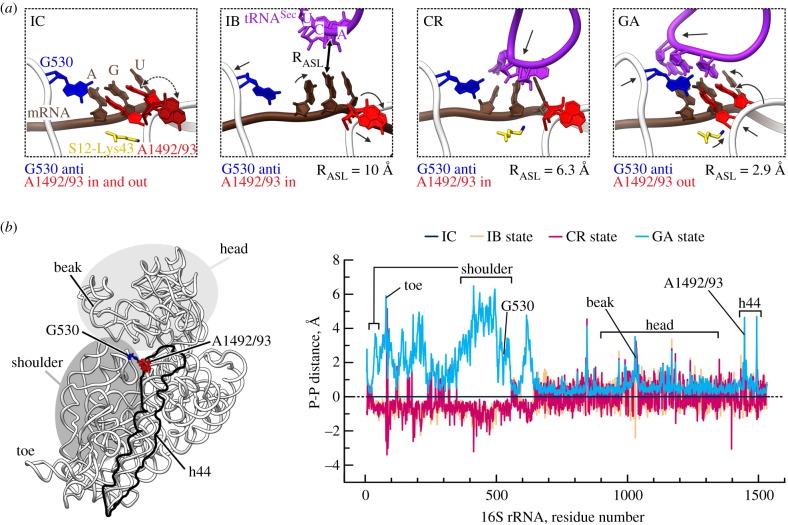
Conformational rearrangements of the SSU upon UGA codon recognition by Sec-tRNA^Sec^ (modified from [13]). (*a*) Local rearrangements of the decoding centre on the SSU. IC, IB, CR and GA are the initial complex prior to SelB-Sec-tRNA recruitment, initial binding, codon reading and GTPase-activated states, respectively. In the IC state, A1492 and A1493 switch between the flipped-in (in) and flipped-out (out) conformations (two-headed arrow); G530 adopts the anti-configuration (anti). (*b*) Global changes of the SSU. Left: 16S rRNA with landmarks indicated; h44, helix 44 of 16S rRNA. Right: Displacements of the 16S rRNA backbone phosphates in the IB, CR or GA states with respect to the IC as obtained by superposition on 16S rRNA; negative values denote domain opening and positive values domain closure of the 30S subunit.

The ternary complex EF-Tu–GTP–aa-tRNA is initially recruited by the C-terminal domain of the multimeric ribosomal protein L12 in an mRNA-independent fashion (figure 1) [30,49]. The rate of initial binding is about 100 µM^−1^ s^−1^ measured both by ensemble kinetics and single molecule fluorescence resonance energy transfer (smFRET) techniques [33,38]. From the initial recruitment of the ternary complex to the L12, aa-tRNA must move towards the SSU where it can sample the codon through reversible excursions of the anticodon in and out of the decoding site [33,37]. From smFRET experiments, the position of the tRNA in the codon reading complex differs from that in the initial binding or the GTPase-activated state, suggesting that the codon reading state is a discrete structural intermediate [38]. Rapid fluctuations at this early stage of decoding are necessary to scan a large number of different ternary complexes before a cognate one is selected. Codon sampling is rapid and is not resolved by ensemble kinetics [34,40]. No structures of the initial binding or codon reading intermediates are available with EF-Tu, consistent with the notion that these complexes are short-lived. We note that although the path for initial recruitment of the EF-Tu- and SelB-ternary complexes is different, with the initial contact involving L12 or SECIS, respectively, the following steps of binding and codon reading are likely to be similar, as in either case the tRNA must scan and recognize the correct codon. Recently, we used cryo-EM and extensive computational sorting to identify intermediates on the trajectory of Sec-tRNA^Sec^ binding to the ribosome, from the initial binding of the ternary complex to tRNA accommodation, and identified two states prior to the formation of the tight codon–anticodon complex. In the initial binding and codon reading intermediates, the SSU is in a more open conformation than in the complex prior to SelB–GTP–Sec-tRNA^Sec^ recruitment [13] (figure 2). This domain opening mainly affects the SSU shoulder, which rotates outwards, away from the LSU (figure 3). The decoding centre remains in an open conformation with both conserved adenines, A1492 and A1493 in a flipped-in (inactive) conformation. SelB and Sec-tRNA^Sec^ span the intersubunit cleft with SelB bound to the shoulder region of the SSU subunit. Because of the SSU domain opening, the GTP-binding domain of SelB does not contact the SRL yet; instead, the tRNA resides on the SRL.

## 4. Codon recognition

Codon recognition (figure 1) was originally identified as a distinct kinetic step leading to an aa-tRNA conformational change and a strong stabilization of the cognate aa-tRNA binding in the complex with EF-Tu and the ribosome [34,40,50]. The stabilization is owing not only to the formation of hydrogen bonds in the codon–anticodon complex (and in some cases to interactions with the natural modifications at the tRNA anticodon loop), but is also caused by the interactions of the codon–anticodon complex with the SSU 16S rRNA. When a cognate aa-tRNA binds to the A site, be it either a canonical aa-tRNA in complex with EF-Tu or Sec-tRNA^Sec^ with SelB, the nucleotides A1492 and A1493 of 16S rRNA change their positions and assume a flipped-out conformation to interact with the second and first positions of the codon–anticodon complex, respectively [13,20] (figures 2 and 3). Because A1492 and A1493 sense the correct Watson–Crick geometry of the base pairs, the local conformational change in the decoding site has a key role in recognition of cognate aa-tRNA.

While it is clear how the cognate aa-tRNA is recognized at the decoding centre of the SSU, it is less clear how structural differences due to mismatches in the codon–anticodon complex result in selectivity and discrimination of incorrect aa-tRNAs. One model is that the near-cognate codon–anticodon complexes are not stabilized to the same degree as the cognate ones and are therefore preferentially rejected. However, comparison of the stabilities of the codon–anticodon complexes in solution [51] and on the ribosome [33,39] suggests that the cognate aa-tRNA is stabilized by about the same factor of approximately 100-fold [19]. Moreover, hydrogen bonds formed between the codon–anticodon complex and the elements of the decoding site of the SSU make a very small contribution to aa-tRNA stabilization and their removal has only modest effects on tRNA selection [52]. Inducing the flipped-out conformation of A1492/1493 with the antibiotic paromomycin, which facilitates the local conformational rearrangement in the decoding centre even in the absence of the cognate aa-tRNA [20], results in a 10- to 20-fold stabilization of aa-tRNA in the codon-recognition state and, importantly, both cognate and near-cognate aa-tRNA binding is stabilized [23]. Thus, somewhat counterintuitively, the stabilizing effect of the ribosome is not specific and as such does not contribute to the discrimination between cognate and near-cognate aa-tRNAs. Nevertheless, steric complementarity in the decoding centre provides the structural basis for the kinetic enhancement of discrimination by induced fit, as described below.

Correct codon–anticodon interaction induces further conformational changes of the complex, including the domain closure of the SSU and distortion of aa-tRNA. Domain closure entails an inward rotation of the SSU shoulder towards the SSU head and the LSU (figure 3). Kinetically, codon–anticodon recognition, rearrangements in the SSU and aa-tRNA distortion occur at the same time and are probably rate-limited by the adjustment of the codon–anticodon duplex in the decoding site. Based on the comparison of the SSU complexes with the cognate and near-cognate ASLs, Ramakrishnan and co-workers [21] suggested that SSU domain closure is a key determinant of tRNA selection. In the following years, the closed SSU conformation was found in a large number of ribosome-bound structures of EF-Tu with cognate aa-tRNA in pre- and post-hydrolysis states [25–27,53–55]. However, high-resolution structures of near-cognate complexes are not available; therefore, the exact details of how mismatches in codon–anticodon complexes affect domain closure remain obscure. Furthermore, some aspects of the domain closure model were questioned based on crystal structures of 70S ribosomes in complex with full-length tRNAs bound to both P and A sites [54]. In particular, A1493 appeared in the flipped-out position also in the absence of the A-site ligand [54,56]. Such differences are generally difficult to explain, as they may stem from the use of 30S subunit versus 70S ribosomes or the absence or the presence of a tRNA in the P site. The recent cryo-EM structures of ribosome-bound SelB–Sec-tRNA complex argue in favour of the conformational mobility of the two adenines and provide a detailed sequence of conformational changes at the SSU upon decoding [13] (figure 3). As long as the A site is not occupied with aa-tRNA, A1492 and A1493 fluctuate from the flipped-in to flipped-out positions even with a tRNA bound in the P site. One such dynamic conformational state might have been captured in the crystal [54]. In the complexes where SelB–Sec-tRNA^Sec^ has bound, but codon–anticodon complex is not yet formed, the SSU is in an open conformation with A1492 and A1493 in the flipped-in (that is inactive) conformation (figure 3) [13]; one can hypothesize that these states are also sampled by non-cognate EF-Tu–aa-tRNA complexes attempting to read the codon. Cognate codon–anticodon interaction facilitates or stabilizes SSU domain closure, and notably, the magnitude of the movement (figure 3) is much larger than that described for the isolated SSU [21]. One can envisage that near-cognate complexes do not induce the same closed conformation and are trapped in an open or partially closed state. The cryo-EM structure of the ribosome-bound EF-Tu with a near-cognate aa-tRNA suggests that the global domain closure did not occur [57]; however, the resolution of that structure might be insufficient to draw the final conclusions.

Knowing the structure of near-cognate aa-tRNA on the ribosome would be a key to understanding the ribosome fidelity. This prompted Yusopova, Yusupov and co-workers [54,55,58] to solve the structures of the ribosome–tRNA complexes with diverse mismatches in the codon–anticodon complex in the A site. However, those complexes turned out to represent snapshots of the ribosome's failure to select a cognate aa-tRNA, rather than pictures of the near-cognate complexes that will be rejected [59]. Because the geometry of such incorrect codon–anticodon complexes is identical to the cognate ones [54,55,58], the ribosome responds by undergoing the same local and global rearrangements as if the complex was a cognate one. The complexes represent those tRNAs that escaped all anticodon complementarity checkpoints and thus show how incorrect tRNA can mimic the correct one to bypass the discrimination mechanisms of the ribosome [59]. It remains unclear whether such codon–anticodon structures can form during initial selection, as they were prepared in the absence of EF-Tu; rather, the complexes may reflect errors of the proofreading step after the release of EF-Tu. Furthermore, while the efficiency of complex formation during crystallization must be very high, in the cell such complexes are extremely rare, which is evident from the very low measured error frequency, which is between 10^−3^ and 10^−8^ [60,61]. Molecular dynamics calculations also showed that tautomerization as such does not cause high codon reading error frequencies, as the resulting tRNA binding free energies are significantly less favourable than for the cognate complex [62]. Thus, the structures of the near-cognate ribosome–tRNA complexes do not tell us how and why incorrect aa-tRNA are selected. Nevertheless, these structures show that the ribosome recognizes the correct geometry of the complex, rather than the number and positions of hydrogen bonds, and demonstrate that from the structural point of view, decoding errors arise when mismatched codon–anticodon duplexes adopt a Watson–Crick shape, which circumvents the discrimination mechanisms of the ribosome [54,55,58].

## 5. GTPase activation

**Figure 4.**
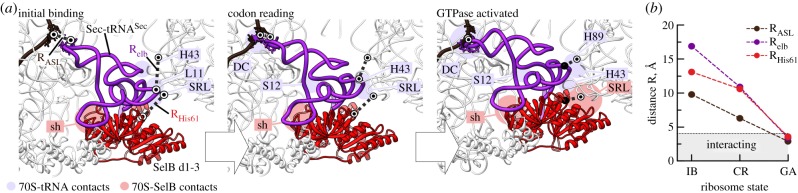
Coupling between Sec-tRNA^Sec^ repositioning, docking of SelB on the SRL and GTPase activation (modified from [13]). (*a*) Sequential docking of SelB-Sec-tRNA^Sec^ on the SRL. Movements of the anticodon (shown by distance denoted as R_ASL_) and elbow (R_elb_ distance) of tRNA^Sec^ and of SelB His61 (R_His61_ distance) are indicated. Ribosome elements interacting with tRNA^Sec^ are shown in mauve, with SelB in pink; sh, 30S shoulder (G357 to U368 of 16S rRNA); S12, protein S12; DC, decoding centre; H43 and H89, helices of 23S rRNA; L11, protein L11. (*b*) Distance changes from state to state; R_ASL_ (N3 of C35 in tRNA^Sec^ to N1 of G in UGA); R_elb_ (tRNA^Sec^ elbow, C5′ of Ψ55 in tRNA^Sec^ to O2′ of A2473 in H89); R_His61_ (ND1 of His61 in SelB to O2′ of G2661 in SRL).

The local changes in the SSU not only stabilize the cognate tRNA in the decoding site, but also initiate transitions that lead to the GTPase activation. Until recently, the mechanism of GTPase activation was not clear. We and others suggested that the tRNA plays a crucial role by helping to induce the catalytically active conformation of EF-Tu. This is supported by crystal structures and biochemical and genetic data suggesting that the integrity, structure and rigidity of the tRNA affect the rate of GTP hydrolysis [26,31,63,64]. In principle, the communication between the decoding centre on the SSU and the GTPase centre of EF-Tu or SelB on the LSU could propagate through tRNA, resulting in a series of conformational changes. However, the recent structures of the ribosome–SelB–GTP–Sec-tRNA^Sec^ complexes suggest a different scenario (figure 4) [13]. We show that codon recognition is communicated by the resulting transition of the decoding site and SSU shoulder domain from an open to a closed conformation which, in turn, moves the tRNA and SelB onto the SRL of the LSU. In the states prior to codon recognition, SelB does not contact the SRL; instead, the SRL interacts with the tRNA, which may even protect the GTPase from premature GTP hydrolysis. GTPase activation occurs upon docking of the GTPase domain of SelB onto the SRL. The docking is assisted by a network of interactions between the SRL and SelB which stabilize the active site conformation of SelB, thereby further facilitating GTP hydrolysis (figure 4) [13]. In the GTPase-activated state, the GTP-binding domain 1 of EF-Tu is bound to the SRL, whereas domain 2 is in contact with the shoulder of the SSU [25–27]. The head and shoulder domains of the SSU are in the closed conformation and the tRNA is strongly distorted. The arrangement of the SSU domains, the contacts of domains 1 and 2 with the SRL and the SSU shoulder and the distortion of the tRNA are very similar for EF-Tu and SelB, suggesting the evolutionary conservation of the GTPase activation mechanism [13].

One remaining question is why the mismatches in the codon–anticodon complex impede GTPase activation. The rate of GTP hydrolysis increases by three to seven orders of magnitude in response to the correct codon–anticodon interaction in the decoding centre [33,34,39,41,65]. Also the smFRET data on decoding suggest that the reactions leading to GTP hydrolysis are more rapid with the cognate than near-cognate aa-tRNA [37,38]. The initial source of the selectivity of GTPase activation must come from the dynamics of the decoding site. This is demonstrated by the finding that inducing the ‘correct’ conformation of the decoding site by paromomycin increases the GTPase rate in the near-cognate ternary complex by a factor of 100, whereas in the cognate complex the rate is even slightly reduced; as a result, near-cognate aa-tRNA is incorporated more efficiently resulting in a higher error frequency [23]. Additionally, the effects of paromomycin on the GTPase activation may be due to its effect on the position of helix 69 of 23S rRNA [54]. The global dynamics of the SSU is crucial, because restricting the conformational flexibility of the SSU by the antibiotic streptomycin alters the rates of GTP hydrolysis in such a way that they became almost identical on cognate and near-cognate codons, resulting in virtually complete loss of selectivity [22]. These results, as well as genetic epistatic analysis of ribosome ambiguity mutation (*ram*) at the h12/S4/S5 region and at the intersubunit bridge B8 [66] are in line with the notion that the SSU domain closure is important. Also the existence of negative regulatory elements in the SSU is consistent with the importance of coordinated, strictly defined motions of the SSU [29]. However, the distortion of the tRNA plays a key role. Mutations in the tRNA D-arm of tRNA^Trp^ accelerate the rates of GTP hydrolysis independently of codon–anticodon pairing [31]. Mutation A9C tRNA^Trp^ accomplishes this by increasing tRNA flexibility, whereas G24A tRNA^Trp^ allows the formation of an additional hydrogen bond that stabilizes the distortion [63]. However, the tRNA is distorted when bound to the ribosome either on cognate or near-cognate codons, as shown both by cryo-EM and fluorescence quenching studies [57,67]; the latter work indicates a somewhat larger distortion of the tRNA in a near-cognate complex. It is difficult to speculate how the structure of the near-cognate GTPase-activated state may look. Codon–anticodon mismatches change the accessible range of global SSU fluctuations, so that EF-Tu does not reach the SRL. The lower rate of GTP hydrolysis may be a result of a misalignment at the active site or of a lower success rate of the fluctuations from the codon recognition to GTPase-activated state.

## 6. GTP hydrolysis

**Figure 5.**
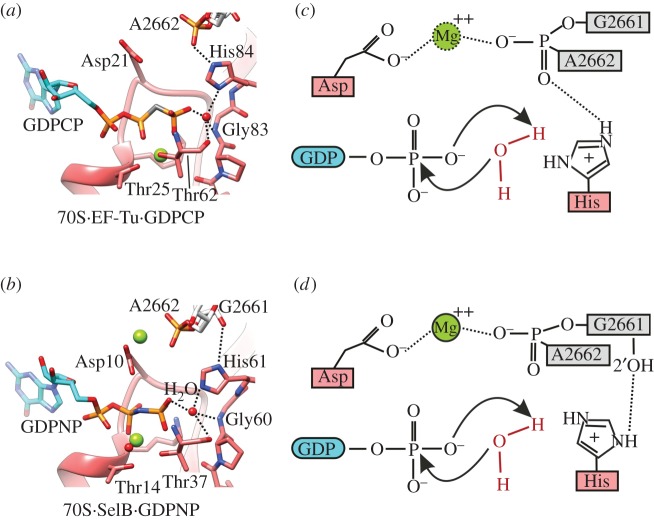
The active site of EF-Tu and SelB and the mechanism of GTP hydrolysis. Close up of the GTPase centre of (*a*) EF-Tu (PDB: 4V5 L) and of (*b*) SelB (PBD: 5LZD) in the activated state (modified from [13]). (*c*) and (*d*) The mechanism of GTP hydrolysis in translational GTPases. Two universally conserved residues, an Asp in the P-loop and a His in the switch II motif contribute to proper positioning of the γ-phosphate of GTP and of the water molecule, respectively. The side chain of the catalytic His is protonated upon contact with the SRL. The role/position of the Mg ion is based on the GTPase-activated pre-hydrolysis structures of EF-G (PDB 4V90, 4JUW, REF) in (*c*) and SelB (PDB 5LZD [13]) in (*d*).

EF-Tu and SelB have a nucleotide binding pocket which is conserved in the majority of translational GTPases [68]. Two universally conserved residues in the Pro-Gly-His (PGH) motif of the switch II region and in the P loop, His84 and Asp21, respectively, in EF-Tu, are crucial for GTP hydrolysis on the ribosome (figure 5) [69]. By contrast, the slow intrinsic GTPase activity of EF-Tu proceeds through a mechanism that is independent of His84 and Asp21. Instead, a monovalent K^+^ ion coordinated by Asp21 has a small stimulatory effect. Upon binding to the ribosome the mechanism of GTPase reaction changes [69–72]. The key ribosome element responsible for the activation is the SRL of the LSU [26,73]. Ribosomal protein L12 also contributes to the GTPase activation through a yet unknown mechanism [49,74]. The rate of GTP hydrolysis is independent of pH, which indicates that neither His84 nor Asp21 acts as a general base or acid catalyst [69,75]; the kinetic solvent isotope effect is small, suggesting that proton transfer in the transition state is not rate-limiting [69]. Both observations are consistent with the computer simulations of the reaction pathway [70–72]. The predicted p*K*_a_ of His84 is shifted upwards so that the sidechain is positively charged at neutral pH [72]. The most probable role for H84 is thus to contribute to catalysis by positioning of the nucleophilic water molecule for attack, which is triggered by the interaction of EF-Tu with A2662 of the SRL upon codon recognition. The conserved Asp21 may contribute to the acceleration of GTP hydrolysis by providing an optimal orientation of the γ-phosphate. Computer simulations suggest a concerted mechanism of hydrolysis with early proton transfer from water to the γ-phosphate group of GTP, followed by nucleophilic attack by hydroxide ion. His84 and the backbone of the PGH peptide act to ‘pull’ at the γ-phosphate negative charge, while Asp21 acts as to ‘push’ it in the same direction [72]. Thus, GTP hydrolysis is largely governed by the electrostatics of the reaction centre [69–72]. The Asp-bound Mg^2+^ ion is not resolved in EF-Tu, but is clearly seen in the ribosome-bound structures of SelB and EF-G (Asp10 and Asp22, respectively) [13,76,77]. Interestingly, some details of interactions at the GTPase active site appear different in EF-Tu and SelB (figure 5). Crystal structures suggest that in EF-Tu (and EF-G) the main chain phosphate oxygen of the SRL A2662 interacts with the NE2 group of His, whereas in SelB the 2′OH of SRL G2661 interacts with ND1 of His. This does not change the overall mechanism, but may in part account for differences in the rates of GTP hydrolysis by EF-Tu and EF-G (more than 100 s^−1^) and SelB (10 s^−1^).

## 7. Accommodation of aa-tRNA in the A site

**Figure 6.**
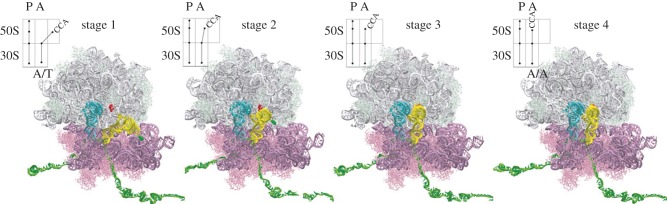
Model of the aa-tRNA accommodation pathway (from [28]; copyright 2005 National Academy of Sciences USA). Stage 1 represents the hypothetic state after the dissociation of EF-Tu, but before the onset of tRNA accommodation. Stage 2 shows the movement of the aminoacyl-tRNA body (except for the 3′CCA portion of tRNA) towards the PTC. In stage 3, the aminoacyl-tRNA body is accommodated. During stage 4, the aminoacyl-tRNA 3′-CCA end is accommodated into the PTC. The schematics depict the process of accommodation.

Accommodation comprises the movement of aa-tRNA after its release from EF-Tu into the peptidyl transferase centre on the LSU [40]. The tRNA CCA end moves by about 100 Å through the so-called accommodation corridor [28]. Molecular dynamics simulations suggest that tRNA accommodation proceeds in three steps, with sequential movements of aa-tRNA elbow, acceptor arm and then 3′-CCA end entry into the PTC (figure 6). Unperturbed accommodation is very efficient, with virtually all of cognate aa-tRNA entering the peptidyl transferase centre and undergoing peptide bond formation [39,40]. However, when peptide bond formation is abolished owing to the presence of a deacylated tRNA in the P site, only 50% of aa-tRNA reaches the final accommodated step as estimated by smFRET experiments [38]. Upon accommodation, aa-tRNA undergoes fluctuations observed as reversible movements of the A-site tRNA elbow region closer to the P-site tRNA [38,78]. These movements likely reflect the first step of accommodation with the elbow of tRNA moving into the close proximity of H89 of LSU [28,44]. Interestingly, smFRET experiments also report on the second step of accommodation, which does not lead to a FRET change but could be extracted from the kinetic analysis of the fluctuations [38]. While the original assignment of this step as peptide bond formation [38] seems unlikely, because all experiments in that work were carried out with a deacylated tRNA in the P site, this step may reflect the accommodation of the acceptor arm and the CCA end. Final adjustment of the CCA end in the PTC may proceed via multiple local pathways owing to the flexibility of the single-stranded CCA end [44].

The biological significance of tRNA accommodation is in providing the mechanism for the aa-tRNA proofreading [39]. When the aa-tRNA is cognate, it is rapidly accommodated [31,33,34,39,79,80]. With a near-cognate aa-tRNA, the rate of accommodation is reduced and the rejection becomes predominant [31,33,34,39,80]. smFRET experiments suggest a similar trend for the accommodation, with the forward fluctuations faster and backward fluctuations slower for the cognate than for near-cognate aa-tRNA [38]. If our assignment of the two accommodation steps observed by smFRET is correct (see above), a 10-fold faster forward movement of the cognate compared to near-cognate aa-tRNA [38] can be interpreted as a crucial role of the acceptor arm accommodation in aa-tRNA discrimination.

Similarly to the initial selection step, the proofreading rates are defined by the codon–anticodon base pairing. The interactions between the cognate codon–anticodon complex and the ribosome elements that resulted in global SSU domain closure during the initial selection step are likely maintained during proofreading, because structures of the complexes in the A/T and A/A states indicate identical interactions in the decoding centre. By contrast, it is less clear whether the rearrangements induced by the near-cognate codon–anticodon complexes are the same or different at the initial selection and proofreading stages; ribosome mutations can differentially affect the aa-tRNA binding stability at the initial selection and proofreading stages [32]. Those near-cognate aa-tRNAs that escaped rejection and have been accommodated into the peptidyl transferase centre adopt geometry mimicking the cognate Watson–Crick base pairs at the first and second codon–anticodon position [54,55,58]. Base pairing at the third position mismatch may adopt unusual geometries, particularly when nucleotide 34 is modified [53,55]. However, the bulk of near-cognate aa-tRNA does not reach the accommodated state. From the structural point of view, the higher rate of near-cognate aa-tRNA rejection may be explained by the reduced stability of the mismatched codon–anticodon complex and the unfavourable geometry imposed by the ribosome, which probably comes at an energetic cost [55]. Whether this leads to the selective destabilization of near-cognate complexes as suggested [55] remains to be tested; as described above, interactions with the decoding site do not selectively destabilize near-cognate codon–anticodon complexes during the initial selection stage [19]. The structural basis for the different accommodation rates of cognate and near-cognate aa-tRNA is not clear, but may result from the misalignment of the near-cognate aa-tRNA [28]. The proofreading step is particularly important when the efficiency of initial selection is low, as it allows reduction of the potentially adverse error of protein synthesis [81]. The remaining errors may be removed by post-transfer editing which employs non-coded termination events [82].

## 8. Concluding remarks

Structural and kinetic work of the past two decades suggested an important contribution of ribosome dynamics, induced fit and kinetic discrimination in the mechanism of decoding and recoding. Recent breakthrough developments in cryo-EM now allow us to follow the ribosome as it selects aa-tRNA in each round of translation. We can now see more clearly how translational GTPases work and how the ribosome discriminates between the correct and incorrect substrates. Knowing the rate constants of decoding by different aa-tRNAs provides the numbers to build predictive models for translation and to explain the existence of natural translational pauses. The mechanism of stop-codon recoding by Sec emerges in great molecular detail. Understanding the structural basis of tRNA selection will require visualizing the analogous sequence of decoding intermediates for EF-Tu-mediated aa-tRNA delivery. Recent work on deciphering the binding pathway of SelB–Sec-tRNA^Sec^ to the ribosome demonstrates that we can now tackle such complex heterogeneous dynamic systems by both structural and biophysical approaches. The ribosome is a dynamic molecular machine; understanding how spontaneous and induced fluctuations of the ribosome and other translational players are rectified into rapid and accurate translation will answer fundamental questions about the movement and selectivity of molecular ensembles.

## References

[1] Bock A 2000 Biosynthesis of selenoproteins—an overview. Biofactors 11, 77–78. (10.1002/biof.5520110122) 10705967

[2] Ambrogelly A, Palioura S, Soll D 2007 Natural expansion of the genetic code. Nat. Chem. Biol. 3, 29–35. (10.1038/nchembio847) 17173027

[3] Caliskan N, Peske F, Rodnina MV 2015 Changed in translation: mRNA recoding by -1 programmed ribosomal frameshifting. Trends Biochem. Sci. 40, 265–274. (10.1016/j.tibs.2015.03.006) 25850333 PMC7126180

[4] Tinoco I Jr, Kim HK, Yan S 2013 Frameshifting dynamics. Biopolymers 99, 1147–1166. (10.1002/bip.22293) 23722586 PMC4011568

[5] Herr AJ, Atkins JF, Gesteland RF 2000 Coupling of open reading frames by translational bypassing. Annu. Rev. Biochem. 69, 343–372. (10.1146/annurev.biochem.69.1.343) 10966462

[6] Samatova E, Konevega AL, Wills NM, Atkins JF, Rodnina MV 2014 High-efficiency translational bypassing of non-coding nucleotides specified by mRNA structure and nascent peptide. Nat. Commun. 5, 4459 (10.1038/ncomms5459) 25041899

[7] Kromayer M, Wilting R, Tormay P, Bock A 1996 Domain structure of the prokaryotic selenocysteine-specific elongation factor SelB. J. Mol. Biol. 262, 413–420. (10.1006/jmbi.1996.0525) 8893853

[8] Gromadski KB, Wieden HJ, Rodnina MV 2002 Kinetic mechanism of elongation factor Ts-catalyzed nucleotide exchange in elongation factor Tu. Biochemistry 41, 162–169. (10.1021/bi015712w) 11772013

[9] Paleskava A, Konevega AL, Rodnina MV 2010 Thermodynamic and kinetic framework of selenocysteyl-tRNASec recognition by elongation factor SelB. J. Biol. Chem. 285, 3014–3020. (10.1074/jbc.M109.081380) 19940162 PMC2823455

[10] Thanbichler M, Bock A, Goody RS 2000 Kinetics of the interaction of translation factor SelB from *Escherichia coli* with guanosine nucleotides and selenocysteine insertion sequence RNA. J. Biol. Chem. 275, 20 458–20 466. (10.1074/jbc.M002496200) 10781605

[11] Rudinger J, Hillenbrandt R, Sprinzl M, Giege R 1996 Antideterminants present in minihelix(Sec) hinder its recognition by prokaryotic elongation factor Tu. EMBO J. 15, 650–657. 8599948 PMC449983

[12] Leibundgut M, Frick C, Thanbichler M, Bock A, Ban N 2005 Selenocysteine tRNA-specific elongation factor SelB is a structural chimaera of elongation and initiation factors. EMBO J. 24, 11–22. (10.1038/sj.emboj.7600505) 15616587 PMC544917

[13] Fischer N et al. 2016 The pathway to GTPase activation of elongation factor SelB on the ribosome. Nature 540, 80–85. (10.1038/nature20560) 27842381

[14] Asahara H, Uhlenbeck OC 2002 The tRNA specificity of *Thermus thermophilus* EF-Tu. Proc. Natl Acad. Sci. USA 99, 3499–3504. (10.1073/pnas.052028599) 11891293 PMC122552

[15] Mittelstaet J, Konevega AL, Rodnina MV 2013 A kinetic safety gate controlling the delivery of unnatural amino acids to the ribosome. J. Am. Chem. Soc. 135, 17 031–17 038. (10.1021/ja407511q) 24079513

[16] Yarus M 1992 Proofreading, NTPases and translation: constraints on accurate biochemistry. Trends Biochem. Sci. 17, 130–133. (10.1016/0968-0004(92)90320-9) 1316651

[17] Wohlgemuth I, Pohl C, Rodnina MV 2010 Optimization of speed and accuracy of decoding in translation. EMBO J. 29, 3701–3709. (10.1038/emboj.2010.229) 20842102 PMC2982755

[18] Kurland CG, Ehrenberg M 1984 Optimization of translation accuracy. Prog. Nucleic Acid Res. Mol. Biol. 31, 191–219. (10.1016/S0079-6603(08)60378-5) 6397771

[19] Rodnina MV, Wintermeyer W 2001 Fidelity of aminoacyl-tRNA selection on the ribosome: kinetic and structural mechanisms. Annu. Rev. Biochem. 70, 415–435. (10.1146/annurev.biochem.70.1.415) 11395413

[20] Ogle JM, Brodersen DE, Clemons WM Jr, Tarry MJ, Carter AP, Ramakrishnan V 2001 Recognition of cognate transfer RNA by the 30S ribosomal subunit. Science 292, 897–902. (10.1126/science.1060612) 11340196

[21] Ogle JM, Murphy FV, Tarry MJ, Ramakrishnan V 2002 Selection of tRNA by the ribosome requires a transition from an open to a closed form. Cell 111, 721–732. (10.1016/S0092-8674(02)01086-3) 12464183

[22] Gromadski KB, Rodnina MV 2004 Streptomycin interferes with conformational coupling between codon recognition and GTPase activation on the ribosome. Nat. Struct. Mol. Biol. 11, 316–322. (10.1038/nsmb742) 15004548

[23] Pape T, Wintermeyer W, Rodnina MV 2000 Conformational switch in the decoding region of 16S rRNA during aminoacyl-tRNA selection on the ribosome. Nat. Struct. Biol. 7, 104–107. (10.1038/72364) 10655610

[24] Marshall RA, Aitken CE, Dorywalska M, Puglisi JD 2008 Translation at the single-molecule level. Annu. Rev. Biochem. 77, 177–203. (10.1146/annurev.biochem.77.070606.101431) 18518820

[25] Fischer N, Neumann P, Konevega AL, Bock LV, Ficner R, Rodnina MV, Stark H 2015 Structure of the *E. coli* ribosome-EF-Tu complex at <3 Å resolution by Cs-corrected cryo-EM. Nature 520, 567–570. (10.1038/nature14275) 25707802

[26] Schmeing TM, Voorhees RM, Kelley AC, Gao YG, Murphy FVT, Weir JR, Ramakrishnan V 2009 The crystal structure of the ribosome bound to EF-Tu and aminoacyl-tRNA. Science 326, 688–694. (10.1126/science.1179700) 19833920 PMC3763470

[27] Voorhees RM, Schmeing TM, Kelley AC, Ramakrishnan V 2010 The mechanism for activation of GTP hydrolysis on the ribosome. Science 330, 835–838. (10.1126/science.1194460) 21051640 PMC3763471

[28] Sanbonmatsu KY, Joseph S, Tung CS 2005 Simulating movement of tRNA into the ribosome during decoding. Proc. Natl Acad. Sci. USA 102, 15 854–15 859. (10.1073/pnas.0503456102) 16249344 PMC1266076

[29] McClory SP, Leisring JM, Qin D, Fredrick K 2010 Missense suppressor mutations in 16S rRNA reveal the importance of helices h8 and h14 in aminoacyl-tRNA selection. RNA 16, 1925–1934. (10.1261/rna.2228510) 20699303 PMC2941101

[30] Kothe U, Wieden HJ, Mohr D, Rodnina MV 2004 Interaction of helix D of elongation factor Tu with helices 4 and 5 of protein L7/12 on the ribosome. J. Mol. Biol. 336, 1011–1021. (10.1016/j.jmb.2003.12.080) 15037065

[31] Cochella L, Green R 2005 An active role for tRNA in decoding beyond codon:anticodon pairing. Science 308, 1178–1180. (10.1126/science.1111408) 15905403 PMC1687177

[32] Zaher HS, Green R 2010 Hyperaccurate and error-prone ribosomes exploit distinct mechanisms during tRNA selection. Mol. Cell 39, 110–120. (10.1016/j.molcel.2010.06.009) 20603079 PMC2947859

[33] Gromadski KB, Daviter T, Rodnina MV 2006 A uniform response to mismatches in codon-anticodon complexes ensures ribosomal fidelity. Mol. Cell 21, 369–377. (10.1016/j.molcel.2005.12.018) 16455492

[34] Gromadski KB, Rodnina MV 2004 Kinetic determinants of high-fidelity tRNA discrimination on the ribosome. Mol. Cell 13, 191–200. (10.1016/S1097-2765(04)00005-X) 14759365

[35] Rudorf S, Thommen M, Rodnina MV, Lipowsky R 2014 Deducing the kinetics of protein synthesis *in vivo* from the transition rates measured *in vitro*. PLoS Comput. Biol. 10, e1003909 (10.1371/journal.pcbi.1003909) 25358034 PMC4214572

[36] Rodnina MV, Pape T, Fricke R, Kuhn L, Wintermeyer W 1996 Initial binding of the elongation factor Tu·GTP·aminoacyl-tRNA complex preceding codon recognition on the ribosome. J. Biol. Chem. 271, 646–652. (10.1074/jbc.271.2.646) 8557669

[37] Blanchard SC, Gonzalez RL, Kim HD, Chu S, Puglisi JD 2004 tRNA selection and kinetic proofreading in translation. Nat. Struct. Mol. Biol. 11, 1008–1014. (10.1038/nsmb831) 15448679

[38] Geggier P, Dave R, Feldman MB, Terry DS, Altman RB, Munro JB, Blanchard SC 2010 Conformational sampling of aminoacyl-tRNA during selection on the bacterial ribosome. J. Mol. Biol. 399, 576–595. (10.1016/j.jmb.2010.04.038) 20434456 PMC2917329

[39] Pape T, Wintermeyer W, Rodnina M 1999 Induced fit in initial selection and proofreading of aminoacyl-tRNA on the ribosome. EMBO J. 18, 3800–3807. (10.1093/emboj/18.13.3800) 10393195 PMC1171457

[40] Pape T, Wintermeyer W, Rodnina MV 1998 Complete kinetic mechanism of elongation factor Tu-dependent binding of aminoacyl-tRNA to the A site of the *E. coli* ribosome. EMBO J. 17, 7490–7497. (10.1093/emboj/17.24.7490) 9857203 PMC1171092

[41] Rodnina MV, Fricke R, Kuhn L, Wintermeyer W 1995 Codon-dependent conformational change of elongation factor Tu preceding GTP hydrolysis on the ribosome. EMBO J. 14, 2613–2619. 7781613 10.1002/j.1460-2075.1995.tb07259.x PMC398375

[42] Kothe U, Rodnina MV 2006 Delayed release of inorganic phosphate from elongation factor Tu following GTP hydrolysis on the ribosome. Biochemistry 45, 12 767–12 774. (10.1021/bi061192z) 17042495

[43] Liu W, Chen C, Kavaliauskas D, Knudsen CR, Goldman YE, Cooperman BS 2015 EF-Tu dynamics during pre-translocation complex formation: EF-Tu.GDP exits the ribosome via two different pathways. Nucleic Acids Res. 43, 9519–9528. (10.1093/nar/gkv856) 26338772 PMC4627077

[44] Whitford PC, Geggier P, Altman RB, Blanchard SC, Onuchic JN, Sanbonmatsu KY 2010 Accommodation of aminoacyl-tRNA into the ribosome involves reversible excursions along multiple pathways. RNA 16, 1196–1204. (10.1261/rna.2035410) 20427512 PMC2874171

[45] Plant EP, Nguyen P, Russ JR, Pittman YR, Nguyen T, Quesinberry JT, Kinzy TG, Dinman JD 2007 Differentiating between near- and non-cognate codons in *Saccharomyces cerevisiae*. PLoS ONE 2, e517 (10.1371/journal.pone.0000517) 17565370 PMC1885216

[46] Budkevich TV et al. 2014 Regulation of the mammalian elongation cycle by subunit rolling: a eukaryotic-specific ribosome rearrangement. Cell 158, 121–131. (10.1016/j.cell.2014.04.044) 24995983 PMC4141720

[47] Kotini SB, Peske F, Rodnina MV 2015 Partitioning between recoding and termination at a stop codon-selenocysteine insertion sequence. Nucleic Acids Res. 43, 6426–6438. (10.1093/nar/gkv558) 26040702 PMC4513850

[48] Fischer N, Paleskava A, Gromadski KB, Konevega AL, Wahl MC, Stark H, Rodnina MV 2007 Towards understanding selenocysteine incorporation into bacterial proteins. Biol. Chem. 388, 1061–1067. (10.1515/BC.2007.108) 17937620

[49] Diaconu M, Kothe U, Schlunzen F, Fischer N, Harms JM, Tonevitsky AG, Stark H, Rodnina MV, Wahl MC 2005 Structural basis for the function of the ribosomal L7/12 stalk in factor binding and GTPase activation. Cell 121, 991–1004. (10.1016/j.cell.2005.04.015) 15989950

[50] Rodnina MV, Fricke R, Wintermeyer W 1994 Transient conformational states of aminoacyl-tRNA during ribosome binding catalyzed by elongation factor Tu. Biochemistry 33, 12 267–12 275. (10.1021/bi00206a033) 7918447

[51] Grosjean HJ, de Henau S, Crothers DM 1978 On the physical basis for ambiguity in genetic coding interactions. Proc. Natl Acad. Sci. USA 75, 610–614. (10.1073/pnas.75.2.610) 273223 PMC411305

[52] Khade PK, Shi X, Joseph S 2013 Steric complementarity in the decoding center is important for tRNA selection by the ribosome. J. Mol. Biol. 425, 3778–3789. (10.1016/j.jmb.2013.02.038) 23542008 PMC3744617

[53] Voorhees RM, Mandal D, Neubauer C, Kohrer C, RajBhandary UL, Ramakrishnan V 2013 The structural basis for specific decoding of AUA by isoleucine tRNA on the ribosome. Nat. Struct. Mol. Biol. 20, 641–643. (10.1038/nsmb.2545) 23542153 PMC3672977

[54] Demeshkina N, Jenner L, Westhof E, Yusupov M, Yusupova G 2012 A new understanding of the decoding principle on the ribosome. Nature 484, 256–259. (10.1038/nature10913) 22437501

[55] Rozov A, Demeshkina N, Khusainov I, Westhof E, Yusupov M, Yusupova G 2016 Novel base-pairing interactions at the tRNA wobble position crucial for accurate reading of the genetic code. Nat. Commun. 7, 10457 (10.1038/ncomms10457) 26791911 PMC4736104

[56] Loveland AB, Bah E, Madireddy R, Zhang Y, Brilot AF, Grigorieff N, Korostelev AA 2016 Ribosome*RelA structures reveal the mechanism of stringent response activation. Elife 5, e17029 (10.7554/eLife.17029) 27434674 PMC4974054

[57] Agirrezabala X, Schreiner E, Trabuco LG, Lei J, Ortiz-Meoz RF, Schulten K, Green R, Frank J 2011 Structural insights into cognate versus near-cognate discrimination during decoding. EMBO J. 30, 1497–1507. (10.1038/emboj.2011.58) 21378755 PMC3102289

[58] Rozov A, Westhof E, Yusupov M, Yusupova G 2016 The ribosome prohibits the G*U wobble geometry at the first position of the codon-anticodon helix. Nucleic Acids Res. 44, 6434–6441. (10.1093/nar/gkw431). 27174928 PMC5291260

[59] Rozov A, Demeshkina N, Westhof E, Yusupov M, Yusupova G 2016 New structural insights into translational miscoding. Trends Biochem. Sci. 41, 798–814. (10.1016/j.tibs.2016.06.001) 27372401

[60] Manickam N, Nag N, Abbasi A, Patel K, Farabaugh PJ 2014 Studies of translational misreading *in vivo* show that the ribosome very efficiently discriminates against most potential errors. RNA 20, 9–15. (10.1261/rna.039792.113) 24249223 PMC3866648

[61] Kramer EB, Vallabhaneni H, Mayer LM, Farabaugh PJ 2010 A comprehensive analysis of translational missense errors in the yeast *Saccharomyces cerevisiae*. RNA 16, 1797–1808. (10.1261/rna.2201210) 20651030 PMC2924539

[62] Satpati P, Aqvist J 2014 Why base tautomerization does not cause errors in mRNA decoding on the ribosome. Nucleic Acids Res. 42, 12 876–12 884. (10.1093/nar/gku1044) PMC4227757 25352546

[63] Schmeing TM, Voorhees RM, Kelley AC, Ramakrishnan V 2011 How mutations in tRNA distant from the anticodon affect the fidelity of decoding. Nat. Struct. Mol. Biol. 18, 432–436. (10.1038/nsmb.2003) 21378964 PMC3072312

[64] Piepenburg O, Pape T, Pleiss JA, Wintermeyer W, Uhlenbeck OC, Rodnina MV 2000 Intact aminoacyl-tRNA is required to trigger GTP hydrolysis by elongation factor Tu on the ribosome. Biochemistry 39, 1734–1738. (10.1021/bi992331y) 10677222

[65] Johansson M, Bouakaz E, Lovmar M, Ehrenberg M 2008 The kinetics of ribosomal peptidyl transfer revisited. Mol. Cell 30, 589–598. (10.1016/j.molcel.2008.04.010) 18538657

[66] Ying L, Fredrick K 2016 Epistasis analysis of 16S rRNA *ram* mutations helps define the conformational dynamics of the ribosome that influence decoding. RNA 22, 499–505. (10.1261/rna.054486.115) 26873598 PMC4793206

[67] Mittelstaet J, Konevega AL, Rodnina MV 2011 Distortion of tRNA upon near-cognate codon recognition on the ribosome. J. Biol. Chem. 286, 8158–8164. (10.1074/jbc.M110.210021) 21212264 PMC3048702

[68] Maracci C, Rodnina MV 2016 Review: Translational GTPases. Biopolymers 105, 463–475. (10.1002/bip.22832) 26971860 PMC5084732

[69] Maracci C, Peske F, Dannies E, Pohl C, Rodnina MV 2014 Ribosome-induced tuning of GTP hydrolysis by a translational GTPase. Proc. Natl Acad. Sci. USA 111, 14 418–14 423. (10.1073/pnas.1412676111) PMC4210003 25246550

[70] Prasad RB, Plotnikov NV, Lameira J, Warshel A 2013 Quantitative exploration of the molecular origin of the activation of GTPase. Proc. Natl Acad. Sci. USA 110, 20 509–20 514. (10.1073/pnas.1319854110) PMC3870692 24282301

[71] Adamczyk AJ, Warshel A 2011 Converting structural information into an allosteric-energy-based picture for elongation factor Tu activation by the ribosome. Proc. Natl Acad. Sci. USA 108, 9827–9832. (10.1073/pnas.1105714108) 21617092 PMC3116401

[72] Aqvist J, Kamerlin SC 2015 The conformation of a catalytic loop is central to GTPase activity on the ribosome. Biochemistry 54, 546–556. (10.1021/bi501373g) 25515218

[73] Wool IG, Gluck A, Endo Y 1992 Ribotoxin recognition of ribosomal RNA and a proposal for the mechanism of translocation. Trends Biochem. Sci. 17, 266–269. (10.1016/0968-0004(92)90407-Z) 1502728

[74] Mohr D, Wintermeyer W, Rodnina MV 2002 GTPase activation of elongation factors Tu and G on the ribosome. Biochemistry 41, 12 520–12 528. (10.1021/bi026301y) 12369843

[75] Daviter T, Wieden HJ, Rodnina MV 2003 Essential role of histidine 84 in elongation factor Tu for the chemical step of GTP hydrolysis on the ribosome. J. Mol. Biol. 332, 689–699. (10.1016/S0022-2836(03)00947-1) 12963376

[76] Tourigny DS, Fernandez IS, Kelley AC, Ramakrishnan V 2013 Elongation factor G bound to the ribosome in an intermediate state of translocation. Science 340, 1235490 (10.1126/science.1235490) 23812720 PMC3836249

[77] Chen Y, Feng S, Kumar V, Ero R, Gao YG 2013 Structure of EF-G-ribosome complex in a pretranslocation state. Nat. Struct. Mol. Biol. 20, 1077–1084. (10.1038/nsmb.2645) 23912278

[78] Polikanov YS et al. 2015 Distinct tRNA accommodation intermediates observed on the ribosome with the antibiotics hygromycin A and A201A. Mol. Cell 58, 832–844. (10.1016/j.molcel.2015.04.014) 26028538 PMC4458074

[79] Ledoux S, Uhlenbeck OC 2008 Different aa-tRNAs are selected uniformly on the ribosome. Mol. Cell 31, 114–123. (10.1016/j.molcel.2008.04.026) 18614050 PMC2709977

[80] Kothe U, Rodnina MV 2007 Codon reading by tRNA^Ala^ with modified uridine in the wobble position. Mol. Cell 25, 167–174. (10.1016/j.molcel.2006.11.014) 17218280

[81] Zhang J, Ieong KW, Mellenius H, Ehrenberg M 2016 Proofreading neutralizes potential error hotspots in genetic code translation by transfer RNAs. RNA 22, 896–904. (10.1261/rna.055632.115) 27090284 PMC4878615

[82] Zaher HS, Green R 2009 Quality control by the ribosome following peptide bond formation. Nature 457, 161–166. (10.1038/nature07582) 19092806 PMC2805954

